# Residues Y142, I143, T146, Q206, S215, R228, G267, and M268 in the surface glycoprotein of FGV-like subgroup A avian leukosis virus are the key sites determining Tva receptor binding and infectivity

**DOI:** 10.1186/s13567-026-01788-w

**Published:** 2026-07-11

**Authors:** Jinqun Li, Qi Li, Yilin Yuan, Biyue Dai, Kangping Lu, Hongyan Pan, Ming Liao, Weisheng Cao

**Affiliations:** 1 College of Veterinary Medicine, South China Agricultural University, Guangzhou, China; 2 Key Laboratory of Zoonosis Prevention and Control of Guangdong Province, Guangzhou, China; 3 National and Regional Joint Engineering Laboratory for Medicament of Zoonosis Prevention and Control, South China Agricultural University, Guangzhou, China; 4 Key Laboratory of Veterinary Vaccine Innovation of the Ministry of Agriculture and Rural Affairs, Guangzhou, China; 5 Key Laboratory of Zoonosis of Ministry of Agriculture and Rural Affairs, Guangzhou, China

**Keywords:** Avian leukosis viruses, Tva, surface glycoprotein, binding affinity, infectivity

## Abstract

Avian leukosis virus subgroup A (ALV-A) invades cells through the specific recognition of the cellular receptor Tva by its encoded surface glycoprotein gp85. Certain Fowl glioma-inducing virus (FGV) strains and their variants belong to ALV-A, but their gp85 proteins differ substantially from classical ALV-A strains. To identify the key residues in the gp85 of FGV-like ALV-A strain 2023ZW001 that determine Tva-binding affinity and infectivity, a strategy of substituting the corresponding gp85 residues between ALV-A 2023ZW001 and ALV-E ev-1 (which uses Tvb as its receptor) was adopted. A series of chimeric gp85 proteins for receptor binding assays were expressed, and multiple recombinant retroviral vectors RCASBP(ZW/ev-1)-EGFP/mCherry were constructed to transfect DF-1 cells for replication capacity analysis. The results showed that host range region 1 (hr1), host range region 2 (hr2), and variable region 3 (vr3) play a decisive role in mediating viral infection. Among these regions, residues 140–146 in hr1 and residues 267–273 in vr3 are the indispensable regions for 2023ZW001 to bind the receptor and mediate viral infection. Residues Y142, I143, and T146 in hr1; residues Q206, S215, and R228 in hr2; and as residues G267 and M268 in vr3 are the key sites determining Tva receptor binding and infectivity. This study indicated that hr1 and hr2 of the FGV-like ALV-A strain contain the major receptor interaction determinants, while vr3 also plays a key role in receptor-binding affinity and infectivity.

**Supplementary Information:**

The online version contains supplementary material available at 10.1186/s13567-026-01788-w.

## Introduction

Avian leukosis (AL), caused by avian leukosis viruses (ALVs), constitutes one of the most devastating viral diseases threatening the global poultry industry [1–3]. As members of the genus *Alpharetrovirus* within the family Retroviridae, ALVs are classified into multiple subgroups (A–K) based on envelope glycoprotein antigenicity, host range (hr), and genetic characteristics [4–7]. Among these subgroups, ALV-A remains prevalent in commercial and indigenous chicken flocks, inducing various neoplastic diseases, severe immunosuppression, and significant economic losses due to reduced egg production, growth retardation, and increased mortality [8, 9].

The successful invasion of ALV into host cells is initiated by the specific interaction between the viral surface glycoprotein gp85 (encoded by the *gp85* gene) and the corresponding cellular receptor, which represents a crucial determinant of viral tropism and pathogenicity [10]. For ALV-A, the Tva protein has been firmly established as the essential cellular receptor. The interaction between ALV-A gp85 and Tva triggers conformational changes in the viral envelope, facilitating the fusion of the viral and cellular membranes and subsequent viral genome entry [11]. The gp85 protein of ALV-A contains several functionally distinct regions, including the host range regions (hr1 and hr2) and variable regions (vr; vr1 to vr3), which have been confirmed to be involved in receptor binding and specificity. Previous studies have identified key residues within these regions of classical ALV-A strains that mediate Tva binding, such as residues 140–142, 199–200, 222–223, and 262, highlighting the structural basis of the ALV-A gp85-Tva interaction [12].

Fowl glioma-inducing virus (FGV) and its variants were initially classified as ALV-A. However, subsequent genomic and phylogenetic evidence has clarified that only a subset of FGV strains truly belong to ALV-A, while the majority of FGV isolates are assigned to other ALV subgroups [13]. Unlike classical ALV-A strains that mainly induce lymphoid tumors, previous FGV-like ALV-A strains from Japan specifically trigger glioma and viral cardiomyopathy in chickens, accompanied by distinct tissue tropism [14, 15]. A notable characteristic of these FGV-like ALV-A strains is the substantial sequence divergence in their gp85 proteins compared with classical ALV-A strains. This sequence variation raises critical questions regarding the molecular mechanisms underlying their interaction with the Tva receptor: whether the key receptor-binding residues identified in classical ALV-A are conserved in FGV-like strains, and if novel determinants exist to compensate for the sequence differences. To date, the structural basis of Tva binding and the determinants of infectivity in FGV-like ALV-A strains remain poorly characterized, which hinders the elucidation of the mechanisms of ALV-A infection and pathogenesis.

The DF-1 cell line used in this study is a continuous fibroblast cell line derived from the CEF line 0, which is sensitive to exogenous ALV but insensitive to endogenous ALV-E [16]. ALV-E uses the Tvb protein as its cellular receptor, which is entirely distinct from the Tva receptor of ALV-A [17]. This clear difference in receptor specificity provides a valuable experimental model for dissecting the receptor-binding determinants of ALV-A gp85. By constructing chimeric gp85 proteins between ALV-A and ALV-E, researchers can precisely map the regions and residues responsible for receptor binding and infectivity [12]. Additionally, the replication-competent avian retroviral vector Replication-Competent Avian Sarcoma-Leukosis Virus Long Terminal Repeat Splice Acceptor Bryan Polymerase (RCASBP) has proven to be a powerful tool for investigating ALV infectivity, as it allows for efficient expression of recombinant viral proteins and quantification of viral entry through reporter genes such as EGFP [18].

This study focused on ALV-A strain 2023ZW001, a newly isolated FGV-like strain, to identify the key residues within its gp85 protein that determine Tva-binding affinity and infectivity in DF-1 cells. A strategy involving the substitution of corresponding gp85 residues between 2023ZW001 and ALV-E strain ev-1 was adopted, complemented by functional assays using chimeric gp85 proteins and RCASBP-based recombinant viral vectors. These findings aim to clarify the molecular basis underlying the interaction between FGV-like ALV-A strains and the Tva receptor, expand understanding of ALV-A genetic diversity and evolution, and lay a foundation for the development of targeted control strategies against these unique ALV-A variants.

## Materials and methods

### Cell cultures and virus

DF-1 cells were cultured in Dulbecco’s Modified Eagle’s Medium (DMEM; Gibco, Thermo Fisher Scientific, USA) supplemented with 10% fetal bovine serum (FBS; Gibco, Australia), 100 U/mL penicillin, and 100 μg/mL streptomycin (Gibco, Thermo Fisher Scientific, USA) at 39 °C in a humidified incubator with 5% CO_2_. 293 T cells were maintained in DMEM containing 10% FBS and cultured at 37 °C in a 5% CO_2_ humidified incubator. FGV-like ALV-A strain 2023ZW001 was isolated from Chinese indigenous chicken flocks, with the GenBank accession number PX712892.

### Sequence analysis

The sequence alignment of the complete genome with other ALV strains was performed using the National Center for Biotechnology Information (NCBI) Basic Local Alignment Search Tool (BLAST) program. Genomic fragments were spliced using SnapGene software. Similarity analysis of different genes of ALV-A 2023ZW001 with other ALV strains were conducted via the Clustal W algorithm implemented in the MegAlign program of DNASTAR Lasergene v7.1 software. A neighbor-joining phylogenetic tree of the *gp85* gene of ALV-A 2023ZW001 was constructed with MEGA 11.0 software, using 1000 bootstrap replicates for statistical support. The GenBank accession numbers and background information of the reference ALV strains are provided in Table 1.

**Table 1:** **ALV reference strains used in this study**

Strains	Subgroup	Accession numbers	Origin	Year
2023ZW001	A	PX712892	China	2024
RSA	A	NC_001408	France	1990
MAV-1	A	L10922	France	1993
MQNCSU	A	DQ365814	UK	2006
TymS_90	A	AB303223	Japan	2007
SDAU09C1	A	HM452339	China	2009
DPRE32	A	KM434201	India	2014
ALV-IBDV2009	A	MT179557	China	2020
YNJC2202	A	PQ476240	China	2022
R-S-R B	B	AF052428	USA	1998
SDAU09C2	B	HM446005	China	2009
Prague C	C	J02342	USA	1977
R-S-R D	D	D10652	Japan	1992
ev-1	E	AY013303	USA	2000
ev-3	E	AY013304	USA	2000
RAV0	E	MF817822	USA	2017
HPRS-103	J	Z46390	UK	1995
ADOL-7501	J	AY027920	USA	2001
JS09GY6	J	GU982310	China	2010
JS11C1	K	KF746200	China	2012
Km_5845	K	AB670314	Japan	2010
GDFX0602	K	KP686143	China	2014

### Construction of recombinant RCASBP (ZW/ev-1)-EGFP/mCherry vectors

The construction method of the RCASBP (A)-EGFP/mCherry retroviral vector (an ALV-based replicative RCASBP vector containing the ALV-A RSA *env* gene) has been previously described [12, 18]. Using RCASBP (A)-EGFP/mCherry as a template, reverse polymerase chain reaction (PCR) was performed using primers F1: 5′-GTATAAGACGTAAACGAAGCGTCTCACACCTGGATG-3′ and R1: 5′-CCTGGCTGCTCGAGTAAGTGGACATCAGCTCTTAC-3′ to obtain a linearized vector. Using primers F2: 5′-CACTTACTCGAGCAGCCAGGGAAC-3′ and R2: 5′-GCTTCGTTTACGTCTTATACCTGTC-3′, the ALV-A 2023ZW001 *gp85* gene was amplified by PCR and cloned into the RCASBP (A)-EGFP/mCherry linearized vector to obtain the recombinant viral vector RCASBP-ZW-EGFP/mCherry. A series of *gp85* fragments with substitutions of ALV-A 2023ZW001 and ALV-E ev-1 were amplified by overlapping PCR and cloned into the RCASBP (A)-EGFP/mCherry linearized vector to construct various recombinant RCASBP (ZW/ev-1)-EGFP/mCherry vectors.

### Construction of plasmids expressing recombinant gp85 proteins

The eukaryotic plasmid pCAGGS-Tva-HA-Fc, which encodes the chicken Tva receptor, HA tag, and human IgG-Fc fragment, has been previously reported [19]. Using overlap PCR, a signal peptide “s” responsible for promoting solubility and a 3 × flag tag were fused to the 5′ and 3′ ends of the ALV-A 2023ZW001 *gp85* gene, respectively, and cloned into the *EcoR I* and *Bgl II* sites of pCAGGS to construct the eukaryotic plasmid pCAGGS-s-ZWgp85-flag. Similar to the method used to construct RCASBP (ZW/ev-1)-EGFP/mCherry, residues of 2023ZW001 gp85 were replaced with corresponding ev-1 residues using overlap PCR, and various recombinant pCAGGS-s-gp85-flag plasmids were constructed through homologous recombination.

### Determination of EGFP/mCherry-positive cell percentage and viral titer

Virus propagation was initiated by transfecting plasmids containing retroviral vector in proviral form. DF-1 cells in six-well plates were transfected with 1 µg of RCASBP (ZW/ev-1)-EGFP/mCherry vector using Lipofectamine 3000 reagent (Invitrogen, Carlsbad, CA, USA) and passaged at 2 days post-transfection (dpt). A part of cells was observed at 5 or 7 dpt using a Leica DMI 4000B fluorescence microscope (Leica Microsystems, Wetzlar, Germany), and the percentage of EGFP/mCherry-positive cells was determined by flow cytometry on a Cytoflex flow cytometer (Beckman Coulter, Brea, CA, USA). Another part of cells was passaged for three consecutive times and cultured in 1% FBS DMEM for 7 d. The supernatant was collected and serially diluted, and the 50% tissue culture infectious dose (TCID_50_) was determined according to the Reed–Muench method.

### Receptor blocking assay

To assess the binding affinity of the recombinant gp85 protein series to the Tva receptor, 5 μg of the recombinant pCAGGS-s-gp85-flag plasmid was transfected into 293 T cells with 95% confluence in a 60 mm culture dish plates using Lipofectamine 3000 reagent. At 2 dpt, the cell culture supernatant was harvested as the source of recombinant gp85 proteins. DF-1 cells with 75% confluence in 24-well plates were rinsed with phosphate-buffered saline (PBS) and incubated with the recombinant gp85 protein solution at 4 °C for 1 h to block the cell-surface Tva receptors. After discarding the supernatant, the DF-1 cells were inoculated with RCASBP-ZW-EGFP at a multiplicity of infection (MOI) of 0.1 for 2 h at 39 ℃. The cells were then washed thoroughly with PBS for five times and maintained in DMEM supplemented with 1% FBS for 5 d. The percentage of EGFP-positive cells was quantified by fluorescence-activated cell sorting (FACS). The higher binding affinity of the gp85 protein to the Tva receptor correlates with the lower percentage of EGFP-positive cells.

### Co-immunoprecipitation (co-IP)

 293 T cells in 60 mm culture dishes were transfected with 5 μg of pCAGGS-s-gp85-flag plasmid using Lipofectamine 3000 reagent. At 2 dpt, cells were lysed with NP-40 buffer (Beyond Biotech, Shanghai, China), and the protein supernatant was collected. The clear supernatant harvested from the pCAGGS-Tva-HA-Fc transfected 293 T cells was concentrated to 1/10 volume using a 10 kDa molecular weight cutoff ultrafiltration rotating column (Merck, Darmstadt, Germany), and the Tva recombinant proteins were purified by gentle stirring with 20 μL of Protein A/G magnetic beads at 4 °C for 2 h. After washing three times with ice-cold PBS, the Protein A/G magnetic beads were incubated with recombinant gp85 protein at 4 °C for 5 h. After washing again with ice-cold PBS, the co-precipitated proteins were denatured by heating at 100 °C for 5 min using sodium dodecyl sulfate–polyacrylamide gel electrophoresis (SDS-PAGE) loading buffer.

### Western blotting

The denatured proteins were separated by 10% SDS-PAGE (Beyotime, Shanghai, China) and transferred onto a nitrocellulose (NC) membrane (Millipore, Burlington, MA, USA). After blocking with 5% bovine serum albumin (BSA, Sigma-Aldrich, St. Louis, MO, USA) in Tris-buffered saline with Tween 20 (TBST) for 1 h at room temperature, the NC membrane was incubated overnight at 4 °C with rabbit anti-HA, anti-flag, and anti-GAPDH monoclonal antibodies (mAbs; ABclonal Biotechnology Co., Ltd., Wuhan, China). Following three washes with TBST, the NC membrane was incubated with DyLight 680-conjugated goat anti-rabbit IgG (H + L) antibody (Abbkine, Redlands, CA, USA) at room temperature for 1 h. The immunoreactive bands were visualized by scanning the NC membrane using an Odyssey infrared imaging system (LI-COR Biosciences, Lincoln, NE, USA).

### Statistical analysis

All data are presented as the mean ± standard deviation (SD) from a minimum of three independent experiments, with each experiment consisting of three technical replicates and were analyzed by Student’s *t*-test using GraphPad Prism 10. A *P*-value of < 0.05 was considered significant. ^*^, ^**^, ^***^, and ^****^ indicate *P*-values less than 0.05, 0.01, 0.001, and 0.0001, respectively.

## Results

### Sequences analysis of FGV-like ALV-A strain 2023ZW001

**Figure 1:**
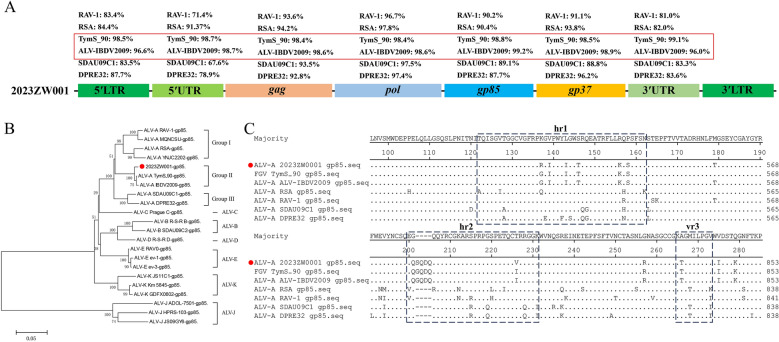
**Sequence analysis of the FGV-like ALV-A strain 2023ZW001. A** Sequence similarity analysis of different genomic fragments of 2023ZW001 with reference ALV-A strains. **B** Neighbor-joining phylogenetic tree of the *gp85* gene of 2023ZW001 and other ALV strains, constructed in MEGA 11.0 software with 1000 bootstrap replicates. 2023ZW001 clusters into Group Ⅱ of ALV-A with TymS_90 and ALV-IBDV2009. **C** Amino acid sequence alignment of gp85 from 2023ZW001 and reference ALV-A strains. Differences are mainly concentrated in the hr1, hr2, and vr3 regions.

Whole-genome nucleotide sequence BLAST analysis revealed that the FGV-like ALV-A strain 2023ZW001, isolated from Chinese indigenous chicken flocks, shared the highest homology (98.4%) with the ALV-A variant FGV prototype strain TymS_90 and the FGV-like strain ALV-IBDV2009, while its homology with classic ALV-A strains was below 94%. Sequence analysis showed that different genomic fragments of 2023ZW001 had the highest similarity to both TymS_90 and ALV-IBDV2009, all of which were no less than 96% (Figure 1A). The sequence homology of *gp85* of 2023ZW001 with the ALV-A reference strain ranges from 87.7% to 99.2%, while the homology with other ALV subgroups ranges from 48.9% to 86.8%. Phylogenetic analysis revealed that these ALV-A strains clustered into three distinct evolutionary clades. Specifically, the *gp85* of 2023ZW001, which exhibits higher identity with that of TymS_90 and ALV-IBDV2009, was grouped into a clade designated Group Ⅱ (Figure 1B). Sequence analysis showed that the amino acid residues of 2023ZW001 gp85 were basically consistent with TymS_90 and ALV-IBDV2009, but significantly different from that of classic ALV-A strains, and these differences were mainly concentrated in the hr1, hr2 and vr3 regions (Figure 1C). The above results indicate that 2023ZW001 is an FGV-like ALV-A strain.

### Reduced replication capacity of recombinant RCASBP virus with RSA gp85 substituted by 2023ZW001 gp85

**Figure 2:**
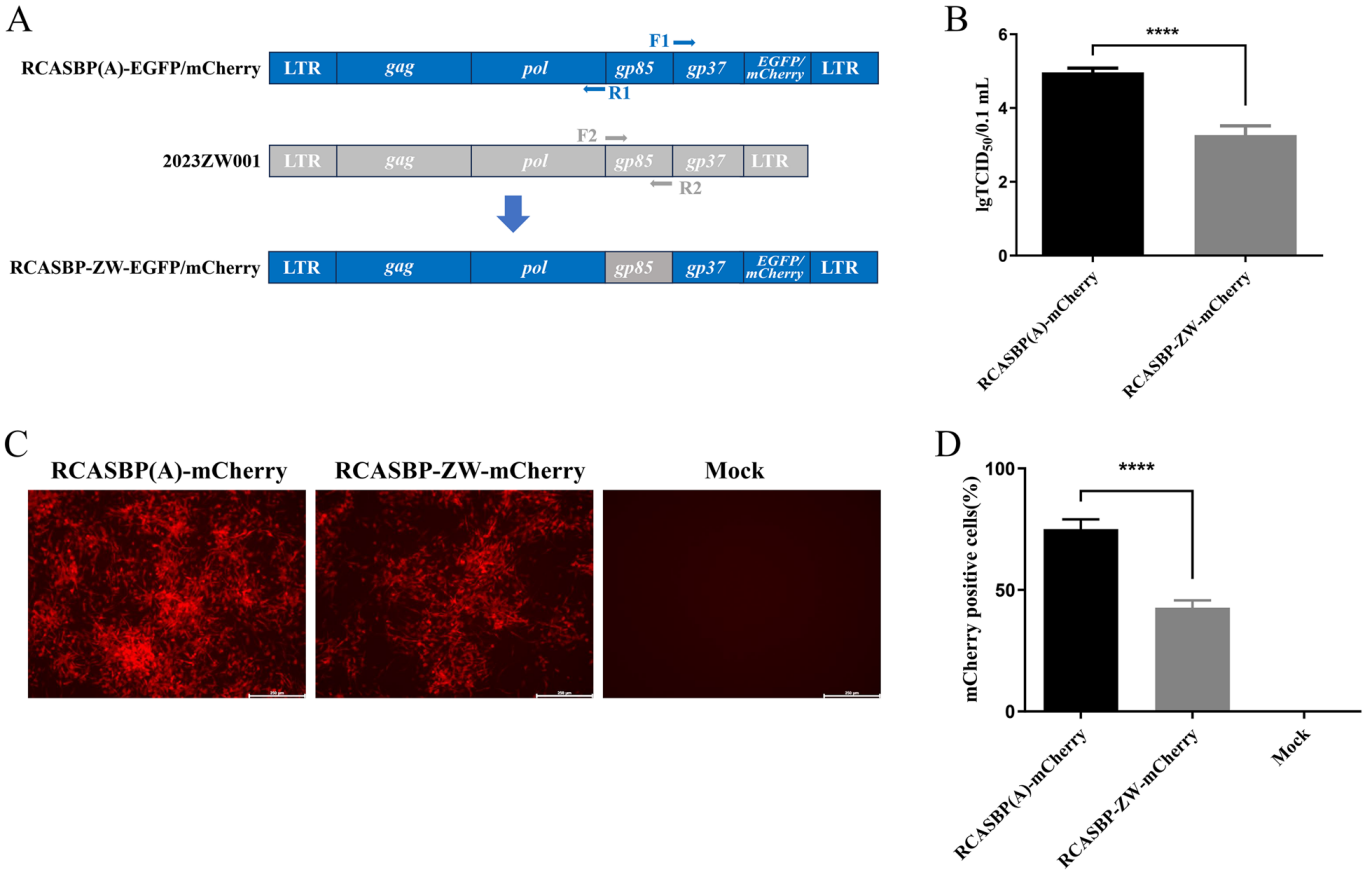
**Reduced replication capacity of recombinant RCASBP virus harboring 2023ZW001 gp85. A** Schematic diagram of the recombinant vector RCASBP-ZW-EGFP/mCherry, constructed by replacing RSA *gp85* in RCASBP(A)-EGFP/mCherry with 2023ZW001 *gp85*. **B** TCID_50_ determination of RCASBP-ZW-mCherry and RCASBP(A)-mCherry in DF-1 cells. **C** Fluorescence microscopy images of DF-1 cells transfected with the two recombinant vectors at 5 days post-transfection (dpt). **D** Flow cytometry analysis of mCherry-positive cell percentages in transfected DF-1 cells at 5 dpt. Data are presented as mean ± SD. ^****^P < 0.0001.

To investigate the infectivity of the FGV-like ALV-A strain 2023ZW001, the recombinant retroviral vector RCASBP-ZW-EGFP/mCherry was constructed by replacing the ALV-A RSA *gp85* gene in the parental RCASBP(A)-EGFP/mCherry (Figure 2A). The TCID_50_ (Figure 2B), fluorescence observation (Figure 2C), and flow cytometry analysis (Figure 2D) results showed that the replication capacity of RCASBP-ZW-mCherry was significantly weaker than that of RCASBP(A)-mCherry in DF-1 cells, suggesting that the amino acid residues determining infection on gp85 of 2023ZW001 are different from those of the classic ALV-A strains.

### Low homology between 2023ZW001 and ALV-E ev-1 in the hr1, hr2, and vr3 regions of gp85

**Figure 3:**
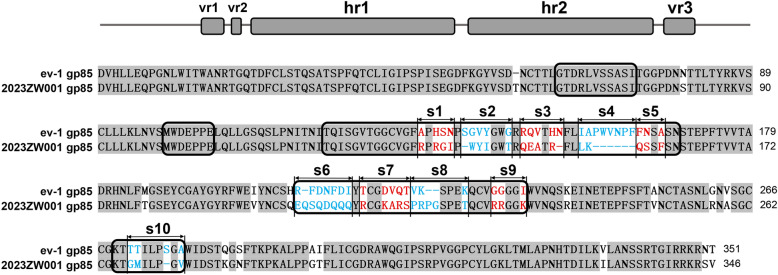
**Schematic diagram of gp85 sequence alignment and segment division between 2023ZW001 and ALV-E ev-1.** The amino acid sequences were aligned via Clustal W method in the MegAlign program. The residues from hr1, hr2, and the vr3 region were divided into ten segments (designated s1–s10).

To identify key residues of 2023ZW001 gp85 that determine infectivity and Tva receptor binding affinity, gp85 of ALV-E ev-1, which uses Tvb as the cellular receptor, was selected as a suitable experimental model. The gp85 of 2023ZW001 and ev-1 are relatively conserved, with an amino acid homology of 84.0%. The vr1 and vr2 regions are amino-acid-same, while the hr1, hr2, and vr3 regions show significant variation. For ease of subsequent research, the residues in the hr1, hr2, and vr3 regions were divided into ten segments, labeled s1–s10 (Figure 3).

### Identification of functional regions in 2023ZW001 gp85 for infectivity

**Figure 4:**
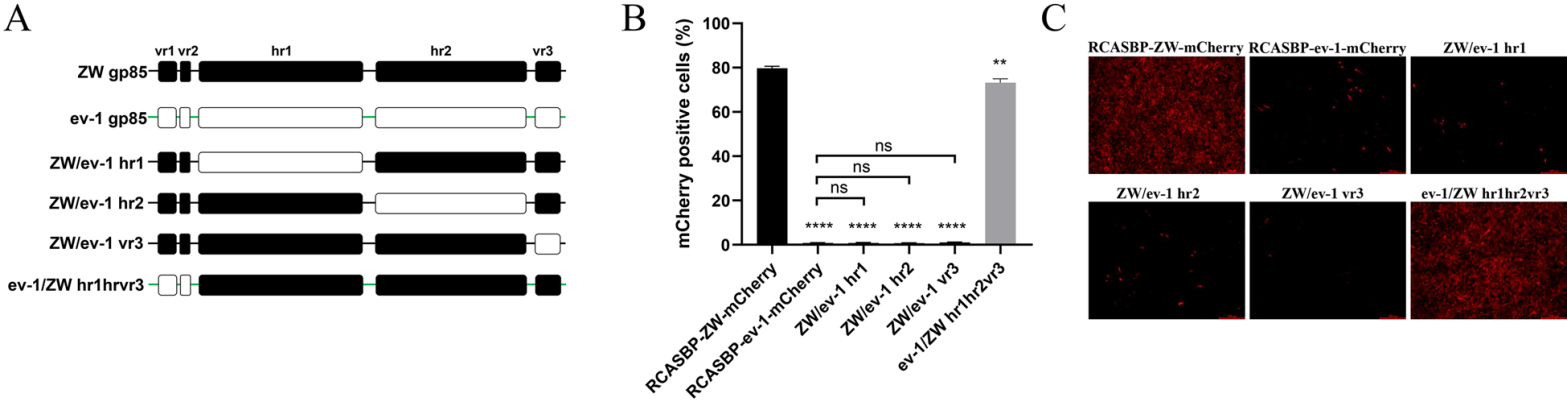
**Identification of functional regions (hr1, hr2, vr3) required for 2023ZW001 infectivity. A** Schematic diagram of chimeric RCASBP(ZW/ev-1)-mCherry vectors, constructed by substituting hr1, hr2, or vr3 between 2023ZW001 and ev-1. **B** Fluorescence microscopy images of DF-1 cells transfected with recombinant vectors at 7 dpt. **C** Flow cytometry analysis of mCherry-positive cell percentages in transfected DF-1 cells at 7 dpt. Data are presented as mean ± SD. *ns* not significant. ^**^P < 0.01; ^****^P < 0.0001.

To verify the functional importance of hr1, hr2, and vr3 for viral infectivity, a series of RCASBP(ZW/ev-1)-mCherry chimeric vectors by substituting these regions between 2023ZW001 and ev-1 gp85 were constructed (Figure 4A). DF-1 cells separately transfected with recombinant vectors were visualized under a fluorescence microscope (Figure 4B) and collected to determine the percentage of mCherry-positive cells by FACS (Figure 4C) at 7 dpt. As expected, replacement of the hr1, hr2, or vr3 region of 2023ZW001 with the corresponding region from ALV-E ev-1 resulted in the complete loss of replication capacity in the chimeric viruses, as the percentage of mCherry-positive cells in these groups showed no significant difference compared to the negative control group (RCASBP-ev-1-mCherry, *P* > 0.05). Conversely, simultaneous replacement of the hr1, hr2, and vr3 regions of ALV-E ev-1 with the corresponding regions from 2023ZW001 gp85 restored the infectivity in DF-1 cells. The above results indicate that hr1, hr2, and vr3 are all necessary elements for the infectivity of 2023ZW001.

### Key segments of 2023ZW001 gp85 for Tva binding and infectivity

**Figure 5:**
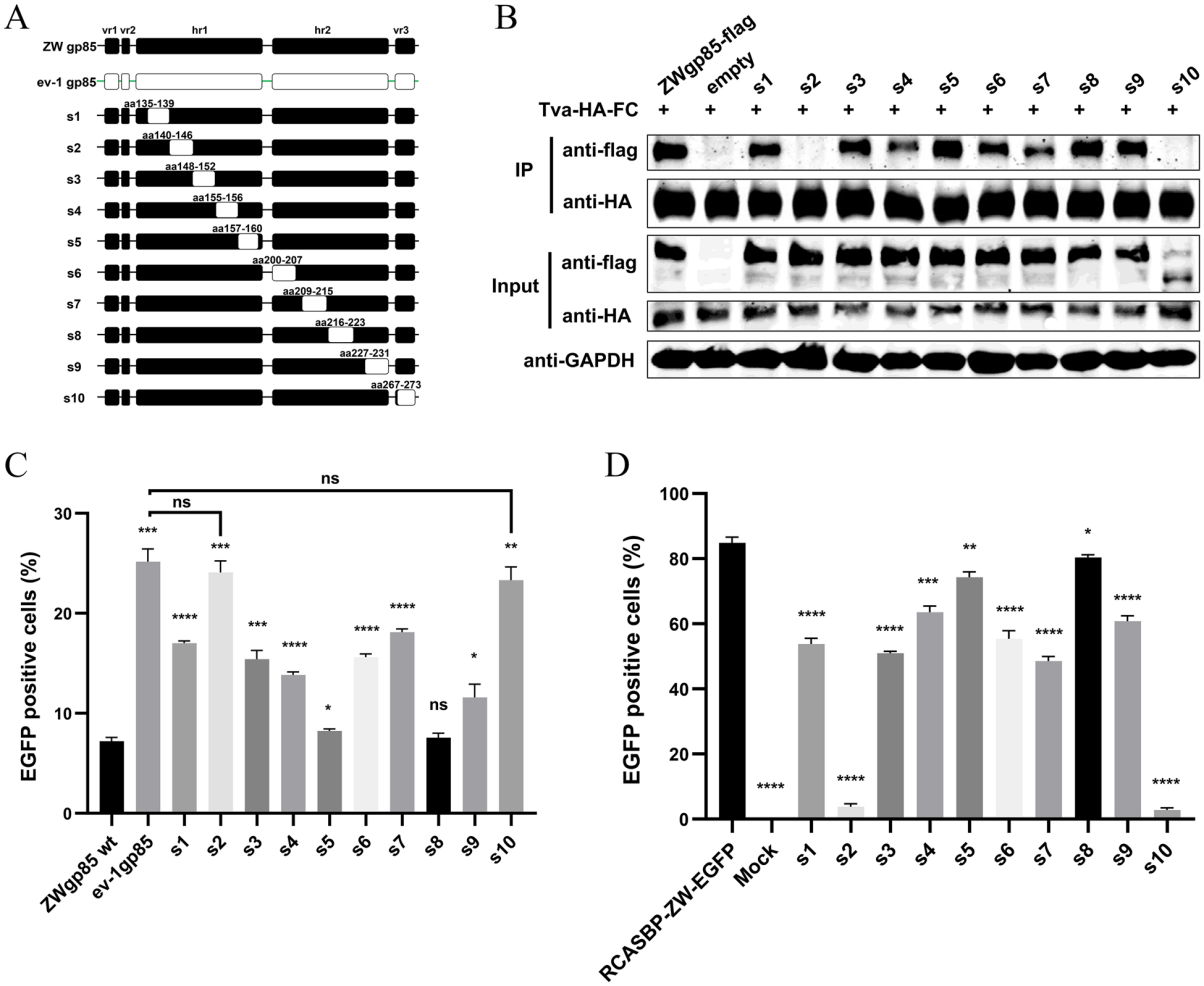
**Identification of key segments in 2023ZW001 gp85 for Tva binding and infectivity. A** Schematic diagram of chimeric gp85 proteins and recombinant RCASBP(ZW/ev-1)-EGFP vectors, constructed by substituting s1–s10 segments between 2023ZW001 and ev-1. **B** Co-IP assay of the interaction between chimeric gp85 proteins and Tva-HA-Fc. **C** Receptor blocking assay. DF-1 cells pre-incubated with chimeric gp85 proteins are infected with RCASBP-ZW-EGFP, and EGFP-positive cell percentages are quantified by flow cytometry at 5 days post-infection (dpi). **D** Flow cytometry analysis of EGFP-positive cell percentages in DF-1 cells transfected with recombinant RCASBP(ZW/ev-1)-EGFP vectors at 7 dpt. Data are presented as mean ± SD.*ns* not significant. ^*^P < 0.05; ^**^P < 0.01; ^***^P < 0.001; ^****^P < 0.0001.

To identify the key segments of 2023ZW001 gp85 for Tva binding and infectivity, hr1, hr2, and vr3 in 2023ZW001 gp85 were divided into ten segments and replaced with the corresponding regions of ev-1 to construct a series of chimeric gp85 proteins and recombinant RCASBP(ZW/ev-1)-EGFP vectors (Figure 5A). Co-IP assays showed that, except for s5 and s8, the substitution of other fragments obviously reduced the binding affinity of recombinant gp85 to Tva receptor. Specifically, the s2 and s10 substituted gp85 protein failed to interact with the Tva receptor, as no specific bands were detected in the immunoprecipitated complexes (Figure 5B). Receptor blocking assays further showed that the recombinant gp85 proteins with s2–s10 substitution all significantly reduced the ability to block RCASBP-ZW-EGFP infection (*P* < 0.05). Moreover, substitution with s2 or s10 resulted in the recombinant gp85 losing its receptor-binding affinity, as the percentage of EGFP-positive cells was similar to that of ev-1 gp85 (Figure 5C). Flow cytometry analysis of the corresponding recombinant viruses showed consistent results, with s2 or s10 substitution leading to complete loss of infectivity, and other segment substitutions all causing significant reduction in the percentage of EGFP-positive cells (Figure 5D; *P* < 0.05). These results indicate that the s2 in hr1 region and s10 (vr3) fragments are indispensable for Tva receptor binding and infectivity.

### Key residues in hr1 region of 2023ZW001 gp85 for Tva binding and infectivity

**Figure 6:**
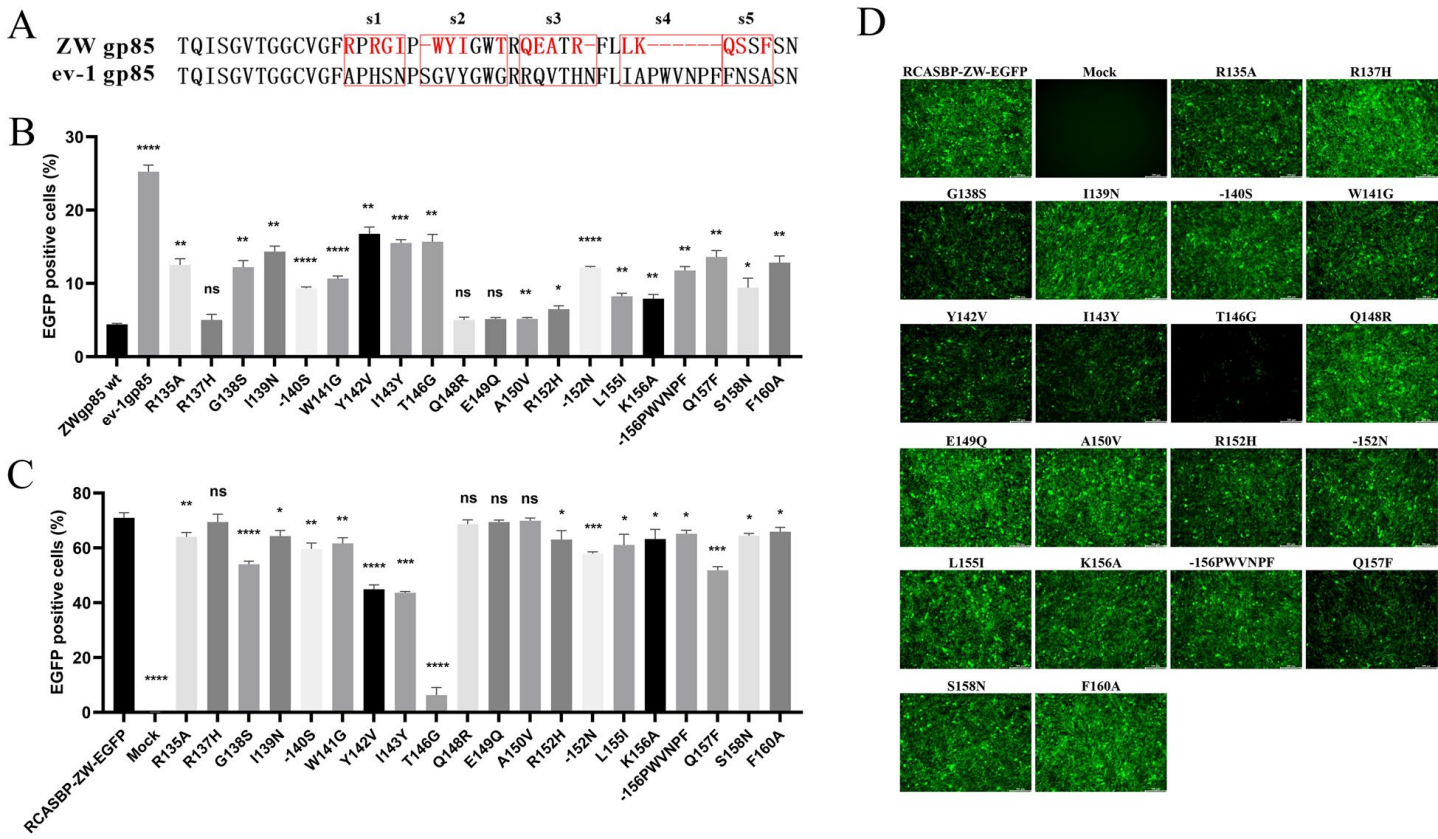
**Identification of key residues in the hr1 region of 2023ZW001 gp85 for Tva binding and infectivity. A** Schematic diagram of point mutants in the hr1 region (divergent residues mutated to ev-1 counterparts). **B** Receptor blocking assay. **C** Flow cytometry analysis of EGFP-positive cell percentages in DF-1 cells transfected with mutant RCASBP(ZW/ev-1)-EGFP vectors at 5 dpt. **D** Fluorescence microscopy images of transfected DF-1 cells at 5 dpt. Data are presented as mean ± SD. *ns* not significant. ^*^P < 0.05; ^**^P < 0.01; ^***^P < 0.001; ^****^P < 0.0001.

To identify the key residues in the hr1 region of 2023ZW001 gp85 determining Tva receptor binding and infectivity, each divergent amino acid in the 2023ZW001 hr1 region was mutated to the corresponding residue of ALV-E ev-1. A series of recombinant gp85 mutant proteins were expressed for receptor blocking assays, and various RCASBP (ZW/ev-1)-EGFP vectors were constructed for viral infection experiments (Figure 6A). The receptor blocking analysis showed that except for the R137H, G148R, E149Q, and A150V mutants, all other recombinant gp85 mutant proteins exhibited a reduced but not fully abrogated blocking effect on viral entry, as their percentages of EGFP-positive cells were significantly higher than that of ZWgp85 wt (*P* < 0.05) but lower than that of ev-1 gp85. Among these mutants, Y142V, I143Y, and T146G showed the most pronounced reduction in receptor blocking efficacy (Figure 6B). As expected, flow cytometry analysis (Figure 6C) and fluorescence microscopy observations (Figure 6D) at 5 dpt with the recombinant retroviral vectors yielded consistent results: the percentage of EGFP-positive cells in the R137H, G148R, E149Q, and A150V mutant groups was not statistically different from that in the RCASBP-ZW-EGFP wt group (*P* > 0.05), whereas all other mutations significantly impaired viral replication in DF-1 cells (*P* < 0.05). Notably, the Y142V, I143Y, and T146G mutations caused the most severe replication defects, with the T146G mutation even rendering the recombinant virus almost completely noninfective. Collectively, these findings demonstrate that Y142, I143, and T146 in the s2 segment of the hr1 region are key residues in 2023ZW001 gp85 that determine Tva receptor binding affinity and viral infectivity.

### Key residues in hr2 region of 2023ZW001 gp85 for Tva binding and infectivity

**Figure 7:**
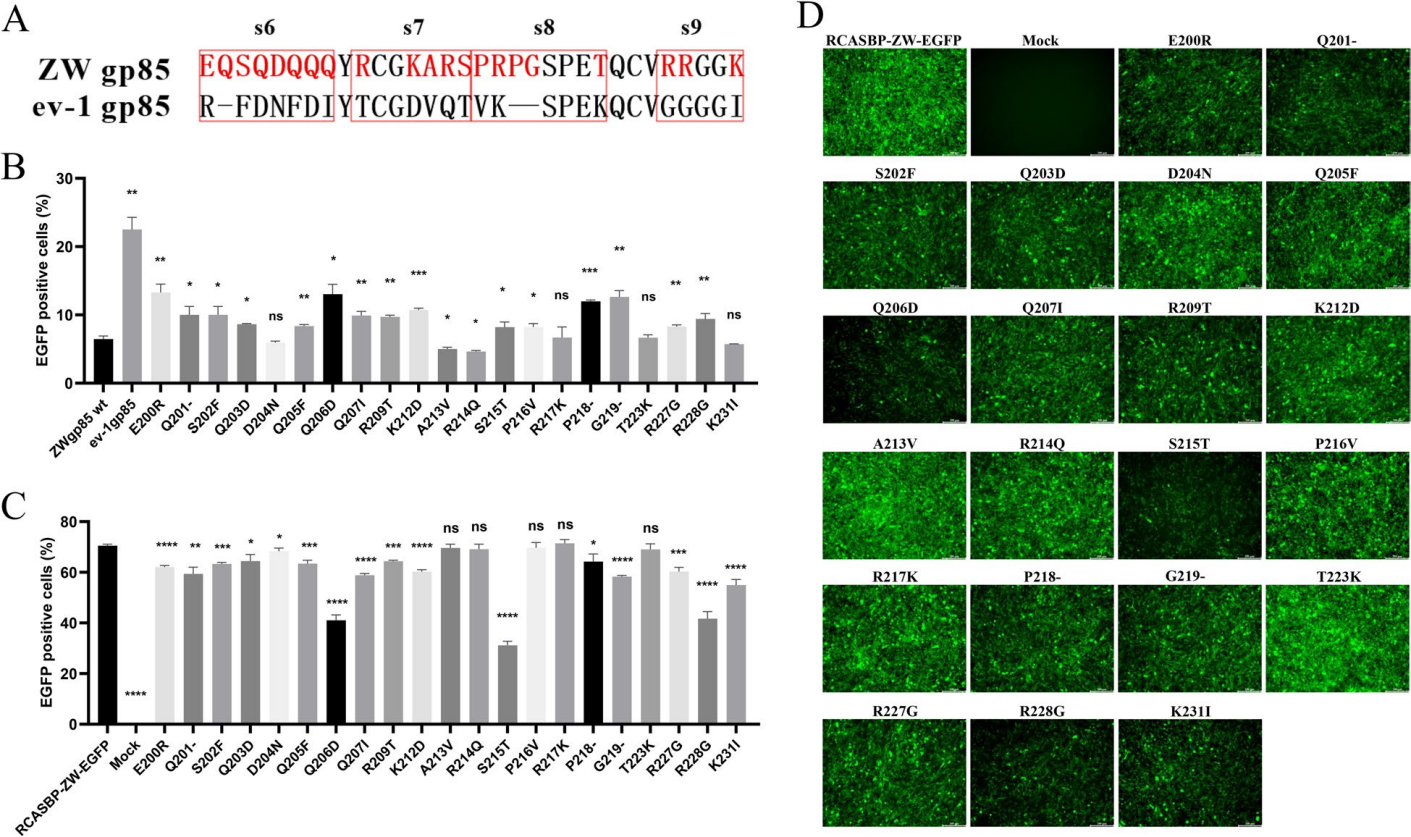
**Identification of key residues in the hr2 region of 2023ZW001 gp85 for Tva binding and infectivity. A** Schematic diagram of point mutants in the hr2 region (divergent residues mutated to ev-1 counterparts). **B** Receptor blocking assay. **C** Flow cytometry analysis of EGFP-positive cell percentages in DF-1 cells transfected with mutant RCASBP(ZW/ev-1)-EGFP vectors at 5 dpt. **D** Fluorescence microscopy images of transfected DF-1 cells at 5 dpt. Data are presented as mean ± SD. *ns* not significant; ^*^P < 0.05; ^**^P < 0.01; ^***^P < 0.001; ^****^P < 0.0001.

To identify the key residues in the hr2 region of 2023ZW001 gp85 for Tva binding and infectivity, a series of point mutants were constructed targeting the divergent amino acid residues between 2023ZW001 and ALV-E ev-1 hr2 region (Figure 7A). Receptor blocking assay results showed that the D204N, R217K, T223K, and K231I mutations had no significant effect on viral entry blocking (*P* > 0.05), while the A213V and R214Q mutations slightly enhanced the blocking efficacy (*P* < 0.05). In contrast, all other mutations significantly impaired the receptor binding capacity (*P* < 0.05; Figure 7B). The infectivity assay results of the recombinant RCASBP (ZW/ev-1)-EGFP vectors were generally consistent with the above findings but with minor discrepancies. Flow cytometry analysis (Figure 7C) revealed that, except for the A213V, R214Q, P216V, R217K, and T223K mutations, all other mutations significantly reduced the percentage of EGFP-positive cells. Among these, the Q206D, S215T, and R228G mutations exerted the most potent inhibitory effects on the infectivity of the recombinant viruses (*P* < 0.0001). Fluorescence microscopy observations were consistent with the flow cytometry results (Figure 7D). Collectively, these findings demonstrate that Q206, S215, and R228 are critical residues in the hr2 region for Tva receptor binding and viral infectivity.

### Key residues in vr3 region of 2023ZW001 gp85 for Tva binding and infectivity

**Figure 8:**
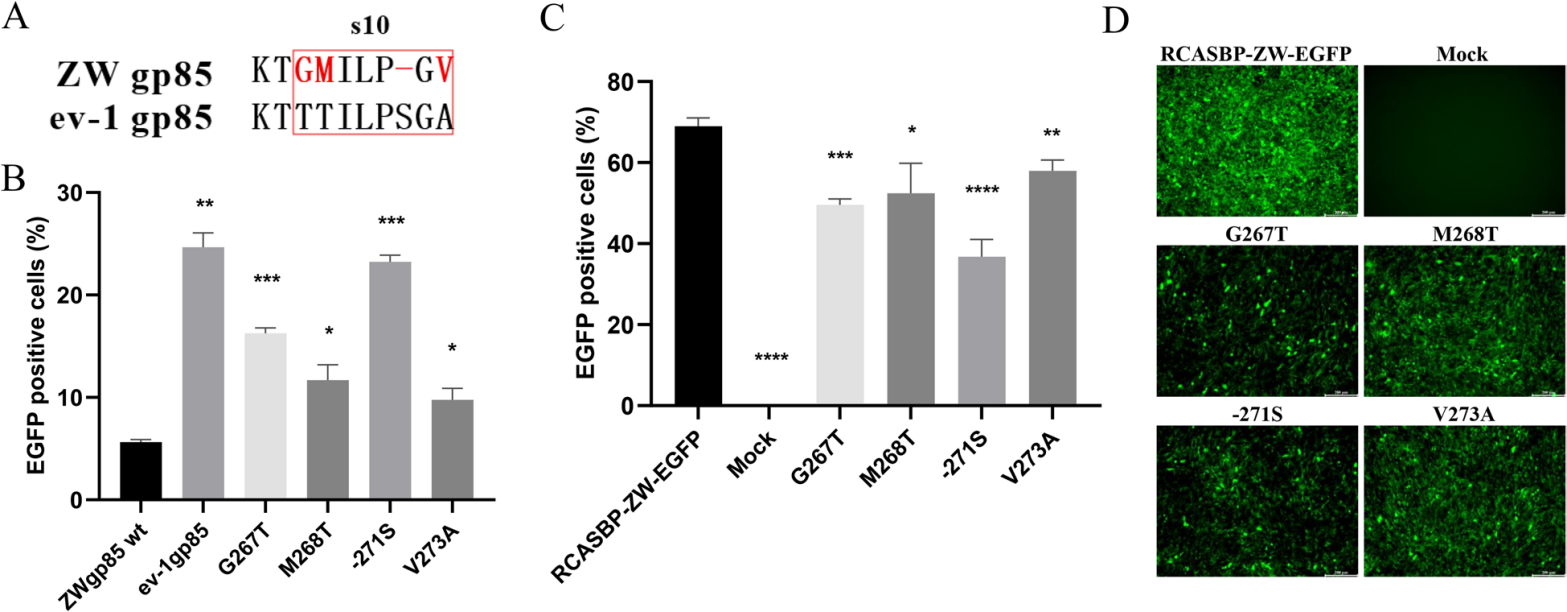
**Identification of key residues in the vr3 region of 2023ZW001 gp85 for Tva binding and infectivity. A** Schematic diagram of point mutants in the vr3 region (divergent residues mutated to ev-1 counterparts). **B** Receptor blocking assay. **C** Flow cytometry analysis of EGFP-positive cell percentages in DF-1 cells transfected with mutant RCASBP(ZW/ev-1)-EGFP vectors at 5 dpt. **D** Fluorescence microscopy images of transfected DF-1 cells at 5 dpt. Data are presented as mean ± SD. *ns* not significant; ^*^P < 0.05; ^**^P < 0.01; ^***^P < 0.001; ^****^P < 0.0001.

To identify the key residues in the vr3 region, a series of point mutants and an insertion mutant were constructed on the basis of the sequence differences in the vr3 region between 2023ZW001 and ALV-E ev-1 (Figure 8A). Receptor blocking assay (Figure 8B) results demonstrated that all mutants significantly impaired viral entry blocking efficacy, with the −271S insertion mutant exhibiting the poorest blocking activity, followed by the G267T and M268T mutants, indicating a marked reduction in their binding affinity to the Tva receptor (*P* < 0.05). Flow cytometry analysis (Figure 8C) revealed that the infectivity of all mutant viruses was diminished, with the residue 271 insertion mutant showing the most severe defect, followed by the G267T and M268T mutants (*P* < 0.05). Fluorescence microscopy images confirmed the impaired replication capacity of these mutants (Figure 8D). Collectively, these findings illustrate that the vr3 region is critical for Tva receptor binding and viral infectivity. Among these residues, G267 and M268 are key determinants, while the insertion at residue 271 severely disrupts the interaction between gp85 and the Tva receptor, thereby abrogating viral entry.

### Restoration of gp85-Tva binding affinity by back mutation

**Figure 9:**
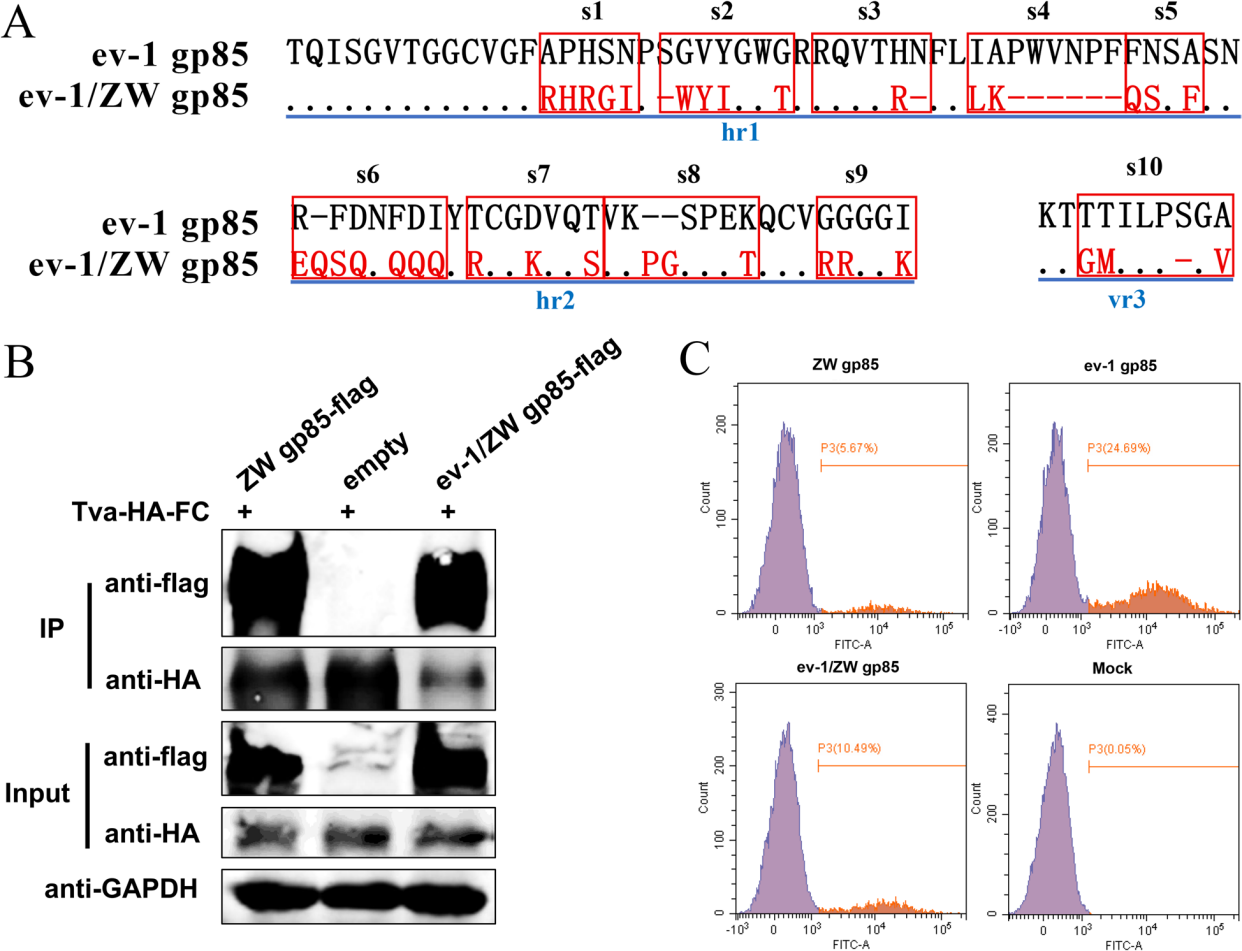
**Restoration of Tva binding affinity by back mutation. A** Schematic diagram of the recombinant ev-1/ZW gp85 protein, constructed by restoring all 2023ZW001 residues that affect infectivity using ev-1 gp85 as a backbone. **B** Co-IP assay of the interaction between recombinant ev-1/ZW gp85 and Tva-HA-Fc. **C** Receptor blocking assay.

To verify the accuracy of the identified residues, a chimeric ev-1/ZW gp85 protein was constructed by replacing all residues that reduce viral infectivity and receptor binding affinity with corresponding residues from 2023ZW001 using ev-1 gp85 as the backbone (Figure 9A). Co-IP assays demonstrated that the chimeric ev-1/ZW gp85 successfully interacted with Tva recombinant protein, albeit with a lower binding affinity compared with the ZWgp85 wt group (Figure 9B). Receptor blocking assays confirmed that the blocking efficacy of the chimeric ev-1/ZW gp85 was lower than that of the ZWgp85 wt but obviously higher than that of ev-1 gp85 (Figure 9C). These results indicate that the identified residues are sufficient to confer the ability of recombinant gp85 protein to bind to Tva receptor, thereby confirming their important roles in mediating the Tva receptor binding and infectivity.

## Discussion

Subgroups A–E avian leukosis viruses (ALVs) are highly homologous retroviruses that evolved the *gp85* gene (encoding surface glycoproteins) from a common ancestor, enabling them to use divergent host proteins as receptors for efficient entry [20]. ALV-A and ALV-K share the Tva receptor [21], while ALV-E shares Tvb with ALV-B/D [17]. Amino acid differences in gp85 between ALV-A and ALV-E are mainly concentrated in three regions (hr1, hr2, and vr3), which are considered the molecular basis of receptor specificity and infectivity [10].

FGV-like ALV-A differ significantly from classical ALV-A in gp85, suggesting distinct molecular mechanisms for Tva receptor binding and infectivity (Figure 1). To verify this, the recombinant RCASBP(A)-mCherry vector with *gp85* gene of ALV-A classical strain RSA substituted by FGV-like strain 2023ZW001 was constructed, and the reduced replication capacity was detected by FACS (Figure 2). To further explore the molecular mechanism of ALV-A FGV-like gp85-mediated receptor binding and infection, a gp85 residue substitution strategy between 2023ZW001 and ALV-E strain ev-1 (consistent with classical ALV-A studies) was adopted (Figure 3). A series of chimeric gp85 proteins were expressed for receptor blocking and co-IP assays, and various retroviral vectors based on RCASBP were constructed to transfect into DF-1 cells—a cell line sensitive to exogenous ALVs but insensitive to ALV-E—for infectivity analysis. The results showed that the substitution of s1 (aa135-139) to s10 (aa267-273) significantly reduced viral blocking efficacy, Tva binding affinity, and replication capacity (Figure 5). Among these, residues Y142, I143, and T146 in hr1, Q206, S215, and R228 in hr2, and G267 and M268 in vr3 were identified as key sites determining receptor binding affinity and infectivity (Figures 6–8). All these eight residues are highly conserved among FGV-like ALV-A strains (Figure 1C). Notably, substitution of these key residues with corresponding ALV-E sequences resulted in complete loss of receptor blocking activity and infectivity (Supplementary Figure 1). Furthermore, the back-mutated ev-1/ZW gp85 showed lower Tva binding affinity than wild-type 2023ZW001 gp85, which may result from the lack of synergistic effects of other auxiliary residues or long-range conformational regulation of gp85 by non-key sites. This study indicates that the hr1, hr2, and vr3 regions of the FGV-like strain 2023ZW001 are collectively indispensable for Tva binding and infectivity, with a molecular basis distinct from classical ALV-A.

Previous retroviral studies classify surface glycoprotein receptor-binding domains into two types: those in hypervariable regions (e.g., murine leukemia virus, ALV-A/B/C/D/E/K) and those consisting of discontinuous segments (e.g., human immunodeficiency virus, equine infectious anemia virus, ALV-J) [10, 19, 22, 23]. This study confirmed that the hr1, hr2, and vr3 regions of 2023ZW001 are collectively essential for Tva binding and infectivity, consistent with our previous findings on ALV-A RSA gp85 [12]. Substitution of any single region with the corresponding ALV-E ev-1 sequence completely abrogated replication in DF-1 cells, while simultaneous substitution of all three regions in ev-1 with those from 2023ZW001 restored Tva binding capacity and infectivity (Figure 4). This finding aligns with previous reports identifying hr1 and hr2 as major receptor-binding domains of ALV-A but further establishes vr3 as a critical determinant, differing from early studies suggesting that vr3 only affects infection specificity without direct involvement in receptor binding.

In-depth analysis via site-directed mutagenesis identified specific key residues in 2023ZW001 gp85 that mediate Tva binding and infectivity, which are significantly different from those identified in RSA gp85 (Supplementary Figure 2) [12]. In the hr1 region of 2023ZW001, mutations at Y142, I143, and T146 significantly reduced receptor binding affinity and viral replication capacity (T146G nearly abolished infectivity; Figure 6), whereas critical hr1 residues of RSA are 140–142 (aa141-143 in 2023ZW001) with W140 (not T146 in 2023ZW001) as the major determinant [12]. This reflects species/strain-specific differences in key residues, a common retroviral evolutionary phenomenon. For example, residue Y142 of 2023ZW001 is essential for chicken Tva binding in DF-1 cells, while Y142 mutation in another ALV-A classical strain SR-A only affects quail Tva binding [24], indicating mutation-specific effects likely reflecting adaptation to host receptor variants. In the hr2 region of 2023ZW001, Q206, S215, and R228 were identified as key residues determining infectivity (Figure 7), whereas key residues of RSA in hr2 are 199–200 (aa200–201 in 2023ZW001) and 222–223 (aa227–228 in 2023ZW001). Additionally, R201D (corresponding to Q206D in 2023ZW001) and S210T (S215T in 2023ZW001) mutations had no effect on receptor binding or infectivity of RSA [12], further confirming distinct molecular bases for gp85-receptor binding between FGV-like and classical ALV-A. For 2023ZW001, vr3 point mutations to ev-1 sequences reduced Tva binding affinity and infectivity, with G267 and M268 identified as key residues. The serine insertion at aa271 caused the most severe impaired function (Figure 8). In contrast, the same insertion at aa266 (aa271 in 2023ZW001) in vr3 of RSA is less inhibitory than that of G262T (G267T in 2023ZW001) [12]. The exact mechanism underlying this functional impairment remains to be further elucidated.

The cellular receptor Tva of ALV-A mediates viral entry via a 40-amino-acid acidic domain homologous to the ligand-binding domain of the low-density lipoprotein receptor (LDLR), which binds ligands through clustered basic residues in the ligand [25, 26]. Sequence analysis of 2023ZW001 gp85 revealed a unique cluster of basic residues in the hr2 region (Figure 1C), suggesting a potential role in receptor recognition. Previous studies indicated that the residues 210, 213, 223, 224, and 227 of the classical ALV-A strain SR-A are critical for efficient infection, with only R213 or K227 alanine substitution reducing Tva binding affinity [27]. While alanine substitution of R223 or R224 in SR-A had no effect on receptor binding, the glycine substitutions of corresponding residues in 2023ZW001 (R227 or R228) significantly reduce viral blocking efficacy and infectivity (Figure 7), indicating the importance of R227 or R228 in FGV-like ALV-A gp85-receptor interaction. In contrast, the R217K mutation (corresponding to R213 in SR-A) had no effect, likely due to the same basicity of lysine (Figure 7). Additionally, the R214Q mutation in 2023ZW001 (corresponding to R209 in SR-A) significantly enhanced receptor blocking efficacy without affecting infectivity, while the K231I mutation (corresponding to K227 in SR-A) did not impact receptor binding affinity but markedly reduced infectivity (Figure 7), indicating inconsistent effects of basic residue mutations on receptor binding and viral invasion. A similar phenomenon was observed for D204N, A213V, and P216V mutations in the hr2 region of 2023ZW001 (Figure 7). Such inconsistent effects of basic residue mutations in hr2 on receptor binding and viral infectivity may be explained by the mechanism that certain basic residues only participate in initial receptor contact but not in post-binding conformational changes required for membrane fusion, while other mutations do not eliminate receptor binding but impair subsequent viral entry steps. These findings reflect the complexity of retroviral envelope–receptor interactions and viral invasion, indicating that identical amino acid mutations in gp85 can exert inconsistent effects on receptor interaction and viral entry.

Due to the low fidelity of reverse transcription, ALVs exhibit an extremely high mutation rate and substantial genetic diversity, enabling rapid adaptation to the external environment [28]. Under external selection pressure, ALVs can alter gp85 structure to adjust entry mechanisms [29]. For example, under selection pressure from ALV-A gp85 immune adhesins, an ALV-A SR-A variant with aa155-160 deletion in hr1 expands receptor usage of Tvb and Tvc while retaining Tva binding [30]. This study employed a site-directed mutagenesis strategy to systematically identify key residues determining receptor interaction and viral entry. However, this strategy cannot truly simulate evolutionary mutations of ALV-A under natural selection pressure, which represents one of the limitations of this study. Since ALV-A and ALV-K share the Tva receptor [11], and ALV-K is widely prevalent in local chicken breeds in East Asia [2], ALV-A gp85 may evolve under selection pressure from ALV-K. Thus, exploring the evolutionary direction of ALV-A gp85 in the presence of ALV-K gp85 immune adhesins represents a valuable research area. Additionally, chimeric substitution between ALV-A and ALV-E gp85 can identify the amino acid residues required for ALV-A infection, whereas identification of the unique residues responsible for the lower replication capacity of the FGV-like strain 2023ZW001 relative to classical ALV-A strain RSA requires reciprocal substitution between these two ALV-A strains. Beyond this, several other research directions are worth exploring to address the study’s limitations. Structural analysis of the 2023ZW001 gp85–Tva complex using techniques such as cryo-electron microscopy will elucidate how the identified residues affect binding affinity and specificity. Additionally, in vivo pathogenicity studies using different ALV-A strains will clarify the associations between key residues, replication capacity, and disease outcomes such as tumor formation and immunosuppression.

## Conclusions

Due to significant amino acid differences in gp85 proteins, the molecular basis underlying gp85-mediated receptor binding and viral infection differs between the FGV-like ALV-A strain 2023ZW001 and classical ALV-A strains. Substitution of s1 (aa135-139) to s10 (vr3 region, aa267-273) in 2023ZW001 with the corresponding segments from ALV-E ev-1 significantly impaired viral entry blocking efficacy, Tva binding affinity, and infectivity; among these regions, s2 (aa140-146) in hr1 and the vr3 regions were identified as essential for these functions. Furthermore, Y142, I143, and T146 in hr1; Q206, S215, and R228 in hr2 and G267 and M268 in vr3 were identified as key residues determining Tva receptor affinity and viral infectivity. This study further confirms that the hr1 and hr2 domains of FGV-like ALV-A harbor the major receptor interaction determinants, while the vr3 domain also plays a crucial role in receptor binding affinity. Collectively, these findings will facilitate a deeper understanding of the infection mechanism of FGV-like ALV-A strains.

## Supplementary Information

** Additional file 1 Effects of key residue substitutions of 2023ZW001 gp85 on receptor blocking activity and viral infectivity. (A) **Receptor blocking assay. DF-1 cells were pre-incubated with recombinant gp85 proteins (wild-type 2023ZW001 gp85, wild-type ev-1 gp85, or recombinant 2023ZW001 gp85 with Y142V, I143Y, T146G, Q206D, S215T, R228G, G267T, and M268T mutations) and then infected with RCASBP-ZW-EGFP. The percentage of EGFP-positive cells was quantified by flow cytometry at 5 days post-infection. (**B**) Fluorescence microscopy images of DF-1 cells transfected with mutant RCASBP(ZW/ev-1)-EGFP vectors at 5 dpt.

**Additional file 2 Comparison of key amino acid residues involved in Tva receptor binding between FGV-like ALV-A strain 2023ZW001 and classical ALV-A strain RSA.** Multiple sequence alignment of the gp85 glycoprotein from 2023ZW001 (red) and representative ALV-A strains, including FGV-like strains TymS-90 and ALV-IBDV2009, as well as classical strains RSA (blue), RAV-1, SDAU09C1, and DPRE32. The conserved majority sequence (Majority) is shown at the top, with functional domains hr1, hr2, and vr3 labeled. Red boxes indicate key residues identified in 2023ZW001 that mediate Tva binding and infectivity (Y142, I143, and T146 in hr1; Q206, S215, and R228 in hr2; G267 and M268 in vr3). Blue boxes highlight the corresponding critical residues in RSA as previously reported (W140, Y141 and L142 in hr1; V199, G200, R222, and R223 in hr2; G262 in vr3). Dots denote identical amino acids relative to the majority sequence and dashes indicate gaps.

## Data Availability

Sequence data that support the findings of this study have been deposited in the NCBI with the primary accession GenBank: PX712892. The data on which the conclusions of the manuscript rely are presented in the main paper.

## References

[1] Qian K, Tian X, Shao H, Ye J, Yao Y, Nair V, Qin A (2018) Identification of novel B-cell epitope in gp85 of subgroup J avian leukosis virus and its application in diagnosis of disease. BMC Vet Res 14:295. 10.1186/s12917-018-1622-x 30257680 10.1186/s12917-018-1622-x PMC6158924

[2] Tan L, Li J, Duan Y, Liu J, Zheng S, Liang X, Fang C, Zuo M, Tian G, Yang Y (2024) Current knowledge on the epidemiology and prevention of avian leukosis virus in China. Poul Sci 103:104009. 10.1016/j.psj.2024.104009 10.1016/j.psj.2024.104009 PMC11298916 39002365

[3] Chen L, Chen X, Liu S, Chen J, Zhang X, Xie Q (2025) Unveiling the host arsenal: interactome profiling of ALV-J p32 integrase reveals novel antiviral targets. Int J Biol Macromol 338:149636. 10.1016/j.ijbiomac.2025.149636 41386616 10.1016/j.ijbiomac.2025.149636

[4] Cui N, Su S, Chen Z, Zhao X, Cui Z (2014) Genomic sequence analysis and biological characteristics of a rescued clone of avian leukosis virus strain JS11C1, isolated from indigenous chickens. J Gen Virol 95:2512–2522. 10.1099/vir.0.067264-0 25009192 10.1099/vir.0.067264-0

[5] Qiao D, He Q, Cheng X, Yao Y, Nair V, Shao H, Qin A, Qian K (2021) Regulation of avian leukosis virus subgroup J replication by Wnt/β-catenin signaling pathway. Viruses 13:1968. 10.3390/v13101968 34696398 10.3390/v13101968 PMC8539648

[6] Xu G, Bei L, Zhao M, Yan H, Zhang H, Jiang S, Zhang R (2025) Isolation and characterization of a recombinant avian leukosis virus subgroup E from a commercial layer farm in Eastern China. Poul Sci 104:105053. 10.1016/j.psj.2025.105053 10.1016/j.psj.2025.105053 PMC11986511 40132316

[7] Chen Q, Shu X, Wang H, Peng Q, Wang W, Su T, Zhou W, Wei K, Zheng X, Li Q, Cao W (2025) THY1 inhibits the replication of ALV—J by diverting chNHE1 for proteasomal degradation. Int J Biol Macromol 322:144816. 10.1016/j.ijbiomac.2025.144816 40451353 10.1016/j.ijbiomac.2025.144816

[8] Zhang R, Mu W, Dong L, Luo S, Zhang S, Yao R, Xu J, Zhang L, Yang L, Xiang B (2025) Molecular characteristics of avian leukosis viruses isolated from indigenous chicken breeds in Yunnan Province, Southwestern China. Poul Sci 104:104850. 10.1016/j.psj.2025.104850 10.1016/j.psj.2025.104850 PMC11810833 39874784

[9] Chen X, Wang X, Yang Y, Fang C, Liu J, Liang X, Yang Y (2022) Enhanced pathogenicity by up-regulation of A20 after avian leukemia subgroup A virus infection. Front Vet Sci 9:1031480. 10.3389/fvets.2022.1031480 36452148 10.3389/fvets.2022.1031480 PMC9702354

[10] Federspiel MJ (2019) Reverse engineering provides insights on the evolution of subgroups A to E avian sarcoma and leukosis virus receptor specificity. Viruses 11:497. 10.3390/v11060497 31151254 10.3390/v11060497 PMC6630264

[11] Přikryl D, Plachý J, Kučerová D, Koslová A, Reinišová M, Šenigl F, Hejnar J (2019) The novel avian leukosis virus subgroup K shares its cellular receptor with Subgroup A. J Virol 93:e00580-19. 10.1128/JVI.00580-19 10.1128/JVI.00580-19 PMC6694804 31217247

[12] Li J, Chen J, Dong X, Liang C, Guo Y, Chen X, Huang M, Liao M, Cao W (2022) Residues 140–142, 199–200, 222–223, and 262 in the surface glycoprotein of subgroup A avian leukosis virus are the key sites determining Tva receptor binding affinity and infectivity. Front Microbiol 13:868377. 10.3389/fmicb.2022.868377 35572683 10.3389/fmicb.2022.868377 PMC9095613

[13] Guo J, Deng Q, Zhu W, Fu F, Liu L, Wei T, Wei P (2023) The phylogenetic analysis of the new emerging ALV-K revealing the co-prevailing of multiple clades in chickens and a proposal for the classification of ALV-K. Front Vet Sci 10:1228109. 10.3389/fvets.2023.1228109 37576830 10.3389/fvets.2023.1228109 PMC10416628

[14] Nishiura H, Tsushima A, Kato A, Saito S, Iwamoto T, Kondo Y, Hatai H, Ochiai K (2023) Avian retroviral cardiomyopathy induced by infectious molecular clones of avian leukosis viruses (fowl glioma-inducing virus variants). Avian Pathol 52:264–276. 10.1080/03079457.2023.2215187 37194644 10.1080/03079457.2023.2215187

[15] Nishiura H, Nakajima T, Saito S, Kato A, Hatai H, Ochiai K (2023) Assessing avian leukosis virus proviral load and lesion correlates in fowl glioma-inducing virus-infected Japanese bantam chickens. J Vet Diagn Invest 35:484–491. 10.1177/10406387231186954 37452573 10.1177/10406387231186954 PMC10467450

[16] Federspiel MJ, Crittenden LB, Provencher LP, Hughes SH (1991) Experimentally introduced defective endogenous proviruses are highly expressed in chickens. J Virol 65:313–319. 10.1128/JVI.65.1.313-319.1991 1845892 10.1128/jvi.65.1.313-319.1991 PMC240519

[17] Adkins HB, Brojatsch J, Young JA (2000) Identification and characterization of a shared TNFR-related receptor for subgroup B, D, and E avian leukosis viruses reveal cysteine residues required specifically for subgroup E viral entry. J Virol 74:3572–3578. 10.1128/jvi.74.8.3572-3578.2000 10729132 10.1128/jvi.74.8.3572-3578.2000 PMC111866

[18] Chen J, Li J, Dong X, Liao M, Cao W (2022) The key amino acid sites 199-205, 269, 319, 321 and 324 of ALV-K env contribute to the weaker replication capacity of ALV-K than ALV-A. Retrovirology 19:19. 10.1186/s12977-022-00598-0 36002842 10.1186/s12977-022-00598-0 PMC9400301

[19] Chen J, Li J, Li L, Liu P, Xiang Y, Cao W (2020) Single amino acids G196 and R198 in hr1 of subgroup K avian leukosis virus glycoprotein are critical for Tva receptor binding. Front Microbiol 11:596586. 10.3389/fmicb.2020.596586 33391214 10.3389/fmicb.2020.596586 PMC7772352

[20] Bova CA, Olsen JC, Swanstrom R (1988) The avian retrovirus env gene family: molecular analysis of host range and antigenic variants. J Virol 62:75–83. 10.1128/JVI.62.1.75-83.1988 2824857 10.1128/jvi.62.1.75-83.1988 PMC250503

[21] Chen Y, Wang S, Li X, Yu M, Liu P, Meng L, Guo R, Feng X, Liu A, Qi X, Li K, Gao L, Pan Q, Zhang Y, Liu C, Cui H, Wang X, Gao Y (2022) Residues L55 and W69 of Tva mediate entry of subgroup a avian leukosis virus. J Virol 96:e0067822. 10.1128/jvi.00678-22 36069550 10.1128/jvi.00678-22 PMC9517704

[22] Battini JL, Danos O, Heard JM (1995) Receptor-binding domain of murine leukemia virus envelope glycoproteins. J Virol 69:713–719. 10.1128/JVI.69.2.713-719.1995 7815534 10.1128/jvi.69.2.713-719.1995 PMC188633

[23] Zhang Y, Yu M, Xing L, Liu P, Chen Y, Chang F, Wang S, Bao Y, Farooque M, Li X, Guan X, Liu Y, Liu A, Qi X, Pan Q, Zhang Y, Gao L, Li K, Liu C, Cui H, Wang X, Gao Y (2020) The bipartite sequence motif in the N and C termini of gp85 of subgroup J avian leukosis virus plays a crucial role in receptor binding and viral entry. J Virol 94:e01232-20. 10.1128/JVI.01232-20 10.1128/JVI.01232-20 PMC7592230 32878894

[24] Holmen SL, Melder DC, Federspiel MJ (2001) Identification of key residues in subgroup A avian leukosis virus envelope determining receptor binding affinity and infectivity of cells expressing chicken or quail Tva receptor. J Virol 75:726–737. 10.1128/JVI.75.2.726-737.2001 11134286 10.1128/JVI.75.2.726-737.2001 PMC113969

[25] Wilson C, Wardell MR, Weisgraber KH, Mahley RW, Agard DA (1991) Three-dimensional structure of the LDL receptor-binding domain of human apolipoprotein E. Science 252:1817–1822. 10.1126/science.2063194 2063194 10.1126/science.2063194

[26] Rong L, Bates P (1995) Analysis of the subgroup A avian sarcoma and leukosis virus receptor: the 40-residue, cysteine-rich, low-density lipoprotein receptor repeat motif of Tva is sufficient to mediate viral entry. J Virol 69:4847–4853. 10.1128/JVI.69.8.4847-4853.1995 7609052 10.1128/jvi.69.8.4847-4853.1995 PMC189298

[27] Rong L, Edinger A, Bates P (1997) Role of basic residues in the subgroup-determining region of the subgroup A avian sarcoma and leukosis virus envelope in receptor binding and infection. J Virol 71:3458–3465. 10.1128/JVI.71.5.3458-3465.1997 9094617 10.1128/jvi.71.5.3458-3465.1997 PMC191492

[28] Matoušková M, Plachý J, Kučerová D, Pecnová Ľ, Reinišová M, Geryk J, Karafiát V, Hron T, Hejnar J (2024) Rapid adaptive evolution of avian leukosis virus subgroup J in response to biotechnologically induced host resistance. PLoS Pathog 20:e1012468. 10.1371/journal.ppat.1012468 39146367 10.1371/journal.ppat.1012468 PMC11349186

[29] Yin X, Melder DC, Payne WS, Dodgson JB, Federspiel MJ (2019) Mutations in both the surface and transmembrane envelope glycoproteins of the RAV-2 subgroup B avian sarcoma and leukosis virus are required to escape the antiviral effect of a secreted form of the Tvb(S3) receptor+. Viruses 11:500. 10.3390/v11060500 31159208 10.3390/v11060500 PMC6630269

[30] Holmen SL, Federspiel MJ (2000) Selection of a subgroup A avian leukosis virus [ALV(A)] envelope resistant to soluble ALV(A) surface glycoprotein. Virology 273:364–373. 10.1006/viro.2000.0424 10915607 10.1006/viro.2000.0424

