# Supplementary figures and images for: Residues Y142, I143, T146, Q206, S215, R228, G267, and M268 in the surface glycoprotein of FGV-like subgroup A avian leukosis virus are the key sites determining Tva receptor binding and infectivity
Source: Vet Res. 2026 Jul 11;57:133. doi: 10.1186/s13567-026-01788-w (PMC13360851; doi:10.1186/s13567-026-01788-w)

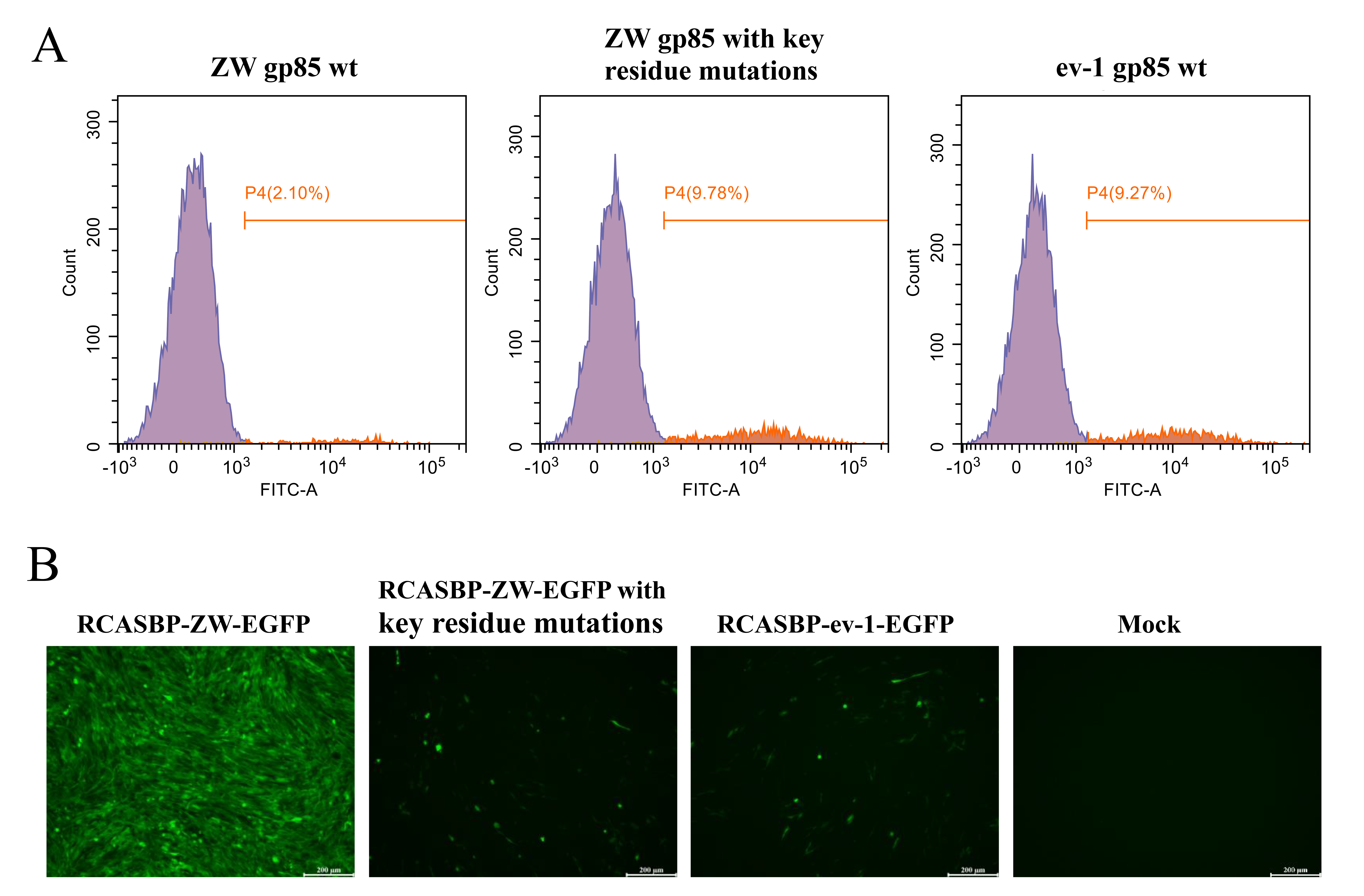

Supplement: Supplementary file 1 — Additional file 1 Effects of key residue substitutions of 2023ZW001 gp85 on receptor blocking activity and viral infectivity. (A) Receptor blocking assay. DF-1 cells were pre-incubated with recombinant gp85 proteins (wild-type 2023ZW001 gp85, wild-type ev-1 gp85, or recombinant 2023ZW001 gp85 with Y142V, I143Y, T146G, Q206D, S215T, R228G, G267T, and M268T mutations) and then infected with RCASBP-ZW-EGFP. The percentage of EGFP-positive cells was quantified by flow cytometry at 5 days post-infection. (B) Fluorescence microscopy images of DF-1 cells transfected with mutant RCASBP(ZW/ev-1)-EGFP vectors at 5 dpt. [file 13567_2026_1788_MOESM1_ESM.tif]

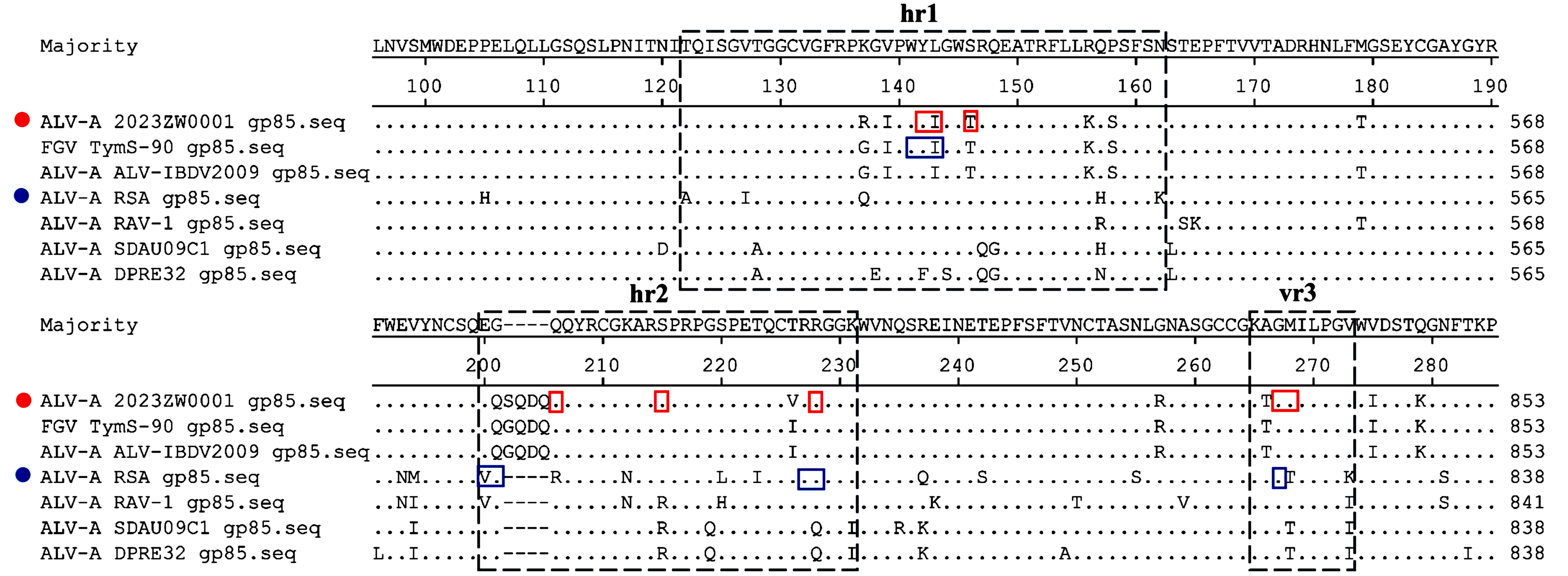

Supplement: Supplementary file 2 — Additional file 2 Comparison of key amino acid residues involved in Tva receptor binding between FGV-like ALV-A strain 2023ZW001 and classical ALV-A strain RSA. Multiple sequence alignment of the gp85 glycoprotein from 2023ZW001 (red) and representative ALV-A strains, including FGV-like strains TymS-90 and ALV-IBDV2009, as well as classical strains RSA (blue), RAV-1, SDAU09C1, and DPRE32. The conserved majority sequence (Majority) is shown at the top, with functional domains hr1, hr2, and vr3 labeled. Red boxes indicate key residues identified in 2023ZW001 that mediate Tva binding and infectivity (Y142, I143, and T146 in hr1; Q206, S215, and R228 in hr2; G267 and M268 in vr3). Blue boxes highlight the corresponding critical residues in RSA as previously reported (W140, Y141 and L142 in hr1; V199, G200, R222, and R223 in hr2; G262 in vr3). Dots denote identical amino acids relative to the majority sequence and dashes indicate gaps. [file 13567_2026_1788_MOESM2_ESM.tif]
